# Associations Between Maternal Prepregnancy Body Mass Index and Gestational Weight Gain and Daughter’s Age at Menarche: The Avon Longitudinal Study of Parents and Children

**DOI:** 10.1093/aje/kwx308

**Published:** 2017-09-11

**Authors:** Rebecca B Lawn, Debbie A Lawlor, Abigail Fraser

**Affiliations:** 1 School of Experimental Psychology, University of Bristol, Bristol, United Kingdom; 2 MRC Integrative Epidemiology Unit, University of Bristol, Bristol, United Kingdom; 3 Department of Population Health Sciences, Bristol Medical School, University of Bristol, Bristol, United Kingdom

**Keywords:** Avon Longitudinal Study of Parents and Children, body mass index, gestational weight gain, menarche, puberty

## Abstract

Earlier puberty and menarche are associated with adverse health outcomes. Reported associations of maternal adiposity with daughter’s age at menarche are inconsistent. We examined associations between maternal prepregnancy body mass index (BMI; weight (kg)/height (m)^2^) and gestational weight gain (GWG) and daughter’s ages at menarche (*n* = 3,935 mother-offspring pairs), pubarche (Tanner stage 2 for pubic hair) (*n* = 2,942 pairs), and thelarche (Tanner stage 2 for breast development) (*n* = 2,942 pairs) in the Avon Longitudinal Study of Parents and Children, a prospective United Kingdom pregnancy cohort study (baseline 1991–1992). During a follow-up period of up to 17 years (1991–2008), mean menarcheal age was 12.6 (standard deviation, 1.2) years. Both maternal prepregnancy BMI and GWG were inversely associated with daughter’s age at menarche after adjustment for maternal age, parity, socioeconomic status, smoking, maternal menarcheal age, and ethnicity (mean differences were −0.34 months (95% confidence interval: −0.45, −0.22) per BMI unit and −0.17 months (95% confidence interval: −0.26, −0.07) per kg, respectively). Associations remained unchanged after adjustment for birth weight and gestational age but were attenuated to the null when results were adjusted for daughter’s prepubertal BMI. Similar results were found for ages at pubarche and thelarche. These findings indicate that greater prepregnancy BMI and GWG are associated with earlier puberty in daughters and that these associations are mediated by daughters’ prepubertal BMIs.

Age at menarche, the start of a woman’s reproductive life, varies both between and within populations (1). Younger age at menarche is associated with premature death (2), breast and ovarian cancer (3, 4), cardiovascular diseases (5), substance abuse (6), depression (7–9), and other adverse outcomes in later life (7–9), while older age at menarche is associated with asthma and poor overall health (10). It is therefore important to identify the determinants of age at onset of puberty and menarche. Research into the developmental origins of reproductive health suggests that the in-utero environment may have long-lasting consequences for the reproductive system (7, 11–14). For example, maternal smoking during pregnancy has been associated with earlier puberty and younger age at menarche (15, 16). Furthermore, greater maternal prepregnancy body mass index (BMI; weight (kg)/height (m)^2^) is likely to be causally associated with greater offspring birth weight and childhood BMI (17), which in turn are associated with earlier puberty (12, 18–21) and younger age at menarche (16). Therefore, it is possible that greater maternal adiposity and gestational weight gain (GWG) are associated with earlier puberty in daughters and that daughters’ own birth weights and/or childhood BMIs mediate this association.

A number of studies have examined the relationship between maternal prepregnancy adiposity and daughter’s menarcheal age, with inconsistent findings. Some authors have reported inverse linear relationships between maternal prepregnancy BMI (13, 22, 23) or GWG (13) and daughter’s age at menarche. Others have reported finding no evidence of associations between maternal BMI or GWG and menarcheal age (23–25). In another study, Boynton-Jarrett et al. (11) found a U-shaped association between GWG and menarcheal age, demonstrating an increased risk of early menarche (defined as <11 years of age) in daughters exposed to extremes of maternal GWG (<10 pounds (<4.5 kg) or >40 pounds (>18.2 kg)). Differing methods of exposure ascertainment (self-reported/directly measured) and/or categorization of GWG and menarcheal age (some studies examined these factors as continuous variables, others in different categories) may account for some of the heterogeneity in findings. Ages at pubarche and thelarche (defined as the appearance of pubic hair and breast development, respectively) have been studied less. In a previous report on the same cohort as that studied here, Maisonet et al. (16) found that girls who entered puberty via a pubarche pathway (i.e., reported pubic hair development before breast development or a combination of the two) were the least likely to have overweight mothers.

We therefore examined the relationships of maternal prepregnancy BMI and GWG with daughter’s age at menarche (primary outcome), age at pubarche, and age at thelarche (secondary outcomes) in a prospective population-based cohort study. We also assessed whether birth weight and/or daughter’s own prepubertal BMI mediated relationships.

## METHODS

### Cohort

The Avon Longitudinal Study of Parents and Children (ALSPAC) is an ongoing prospective, population-based birth cohort study that recruited 14,541 pregnant women resident in Avon, United Kingdom, with expected delivery dates between April 1, 1991, and December 31, 1992 (26, 27). Data collection occurred through self-completed questionnaires or assessment at research clinics. The ALSPAC website contains details on all of the data that are available through a fully searchable data dictionary (28).

**Figure 1.**
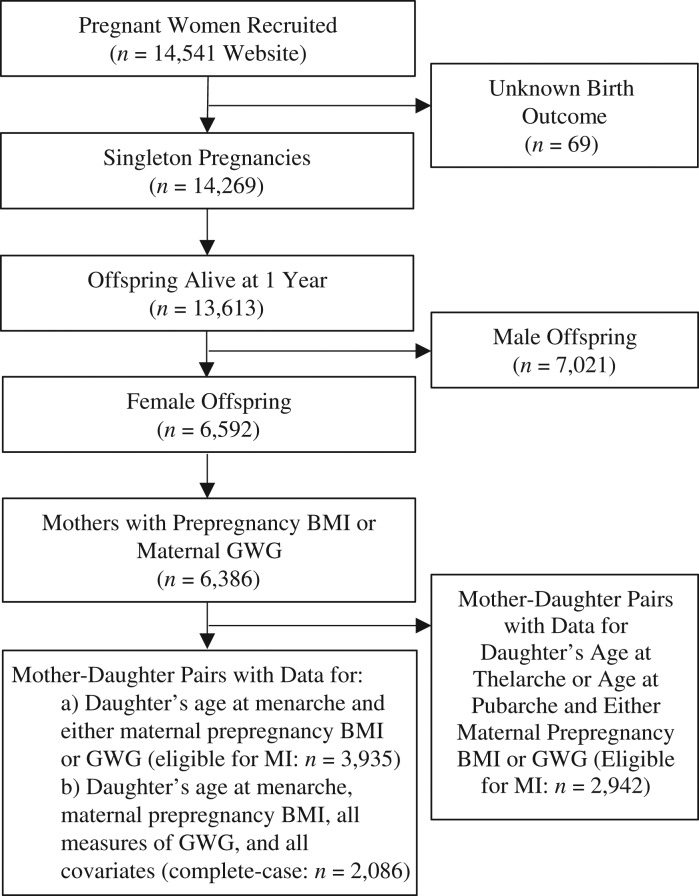
Selection of mother-daughter pairs for an analysis of associations between maternal prepregnancy body mass index (BMI; weight (kg)/height (m)^2^) and gestational weight gain (GWG) and daughter’s ages at menarche, pubarche, and thelarche, Avon Longitudinal Study of Parents and Children, 1991–2008. MI, multiple imputation.

There were 13,613 known mother-offspring pairs with singleton babies who survived to at least 1 year of age; only singleton females were considered in this study (*n* = 6,592). Of these, 3,935 mother-offspring pairs had data on daughter’s menarcheal age and either mother’s prepregnancy BMI or mother’s GWG and were eligible for inclusion (see Figure 1). Follow-up time for the current analysis extended to 17 years from baseline. Ethical approval was obtained from the ALSPAC Law and Ethics Committee and from local research ethics committees.

### Measures

#### Prepregnancy BMI

Mothers reported their immediate prepregnancy weight and height on self-completed questionnaires administered in early pregnancy. This information was then used to calculate prepregnancy BMI, with categories defined as underweight (<18.5), average weight (18.5–24.9), overweight (25.0–29.9), and obese (≥30.0) (29). Reported maternal weight was strongly correlated with measured first antenatal clinic weight (*r* = 0.93).

#### Gestational weight gain

Weight was measured routinely at all antenatal clinic visits at the time of data collection. Six trained midwives abstracted data from obstetrical medical records. Data included every measurement of weight entered and the corresponding gestational age and date for the time of measurement. There was no between-midwife variation in mean values of abstracted data, and repeat data-entry checks demonstrated error rates consistently below 1%. The first weight measurement (kg) was subtracted from the last to determine absolute weight gain, derived for all women who had at least 1 weight measurement prior to 18 weeks’ gestation and 1 after 28 weeks’ gestation. Women were also categorized as having inadequate, adequate, or excessive GWG based on the 2009 Institute of Medicine recommendations (30).

Incremental GWG for term pregnancies only was estimated from a multilevel linear spline model relating weight to gestational age (10 measurements per woman (interquartile range, 8–11)) as previously described (31). This resulted in 4 variables: estimated prepregnancy weight (kg), estimated change in weight between conception and 18 weeks (kg/week), estimated change in weight between 18 and 28 weeks (kg/week), and estimated change in weight between 28 weeks and birth (kg/week) (31).

#### Age at menarche (primary outcome)

Age at menarche was reported by a parent or the participant in a series of postal questionnaires that were distributed approximately annually between the ages of 8 and 17 years, or during research clinic visits made at ages 12.5 and 13.5 years. We then derived menarcheal age as previously described (32). Briefly, we used the first report of age at menarche. If, on a given questionnaire, the respondent indicated that menarche had begun but no age was provided, menarcheal age was estimated as the midpoint between the date of that questionnaire and the previous one (on which the respondent indicated that menarche had not yet begun). This was done only when data from adjacent questionnaires were available (33). If the reported menarcheal age was greater than age at questionnaire completion, age at menarche was coded as missing. Early and late menarche were defined as 1 standard deviation below the mean and 1 standard deviation above the mean, respectively. The remaining participants were categorized as “average” for menarcheal age and were used as a reference group (32).

#### Ages at thelarche and pubarche (secondary outcomes)

In the same series of questionnaires as those for age at menarche, participants were asked about their breast and pubic hair development. For this, participants or their parents were asked to put a tick mark next to the drawing and description that most closely matched the daughter’s current breast and pubic hair development. These drawings corresponded to Tanner stages 1–5 (34). We derived age at thelarche or pubarche as daughter’s age at the midpoint between adjacent questionnaires on which participants had previously reported stage 1 and subsequently reported stage 2 (35).

#### Other variables

Maternal age at delivery was ascertained at enrollment. Daughter’s ethnicity was based on the mother’s reported own ethnicity and the paternal partner’s ethnicity. Parity was reported by the mother and then coded as 0, 1, 2, or ≥3. Maternal menarcheal age was also reported on a questionnaire, with ages below 10 years and above 16 years collapsed. The highest reported parental occupation was used to allocate families to socioeconomic groups (classes 1 (professional/managerial) to 5 (unskilled manual workers)). Information on maternal smoking during pregnancy—categorized as never smoking during pregnancy, smoking before pregnancy or during the first trimester and then stopping (temporarily), or smoking throughout pregnancy—was obtained from questionnaires completed during and after pregnancy.

Gestational age was ascertained from medical records and was based on the date of the last menstrual period, pediatric assessment, obstetrical assessment, or ultrasound assessment. Most commonly, the date of the last menstrual period was used, particularly if the mother felt certain of it and there were no clinical suggestions that it was erroneous. If the date of the last menstrual period was considered unreliable, assessment of the earliest ultrasound measurement was most likely to be used.

Birth weight was obtained from obstetrical records. Daughters’ prepubertal BMIs were assessed during a clinic visit at approximately 7.5 years of age.

### Data analysis

To ascertain whether or not the associations between maternal prepregnancy BMI or GWG and outcomes were linear, we compared models with continuous exposures (in fifths) with models in which exposure fifths were entered as 4 dummy variables. No evidence for departure from linearity was found in any analyses (all *P*’s > 0.1). Linear regression models were then used to examine associations between maternal prepregnancy BMI and GWG (separately) and daughters’ ages at menarche, thelarche, and pubarche. We incrementally adjusted for 1) maternal age only; 2) all confounders: maternal age at pregnancy, ethnicity, parity, maternal age at menarche, socioeconomic status, and smoking during pregnancy (plus gestational age and prepregnancy BMI in GWG models); and 3) all confounders plus potential mediators: daughter’s birth weight (plus gestational age) or prepubertal BMI. We assessed whether associations between maternal prepregnancy BMI or GWG and all outcomes differed according to socioeconomic status, using likelihood ratio tests for interaction testing.

We quantified the natural direct and indirect effects mediated by birth weight (and gestational age) and prepubertal BMI. To obtain indirect effect estimates, we used the difference between the total effect and the natural direct effect. Standard errors were obtained by bootstrapping (100 repetitions). This was done for each imputed data set separately, and results were then combined using Rubin and Schenker’s rules (36).

We used multinomial logistic regression models to examine associations between prepregnancy BMI (as a continuum and categorized as underweight, normal, overweight, or obese), continuous GWG, and categorical GWG (categorized as inadequate, adequate, or excessive based on the 2009 Institute of Medicine criteria (30)) and daughter’s menarcheal age (categorized as early, average, or late). In sensitivity analyses of total GWG and categorical GWG (Institute of Medicine categories), we restricted our sample to term pregnancies. Finally, we examined the association between rates of GWG (prepregnancy weight and weight gain during weeks 0–18, 18–28, and ≥28 of pregnancy, based on knot points at 18 and 28 weeks (37)) and daughter’s ages at menarche, thelarche, and pubarche. We also considered a time-to-event analysis approach; however, there was evidence of departure from proportional hazards in the relationship between maternal prepregnancy BMI and GWG and daughter’s age at menarche (*P* = 0.09 and *P* = 0.08, respectively, based on Schoenfeld residuals).

### Missing data

There were 2,086 mother-daughter pairs with no missing data for exposures, our primary outcome (age at menarche), and all covariables. In order to increase efficiency and minimize bias, we imputed missing values for all mother-offspring pairs with data on daughter’s age at menarche and either maternal prepregnancy BMI or GWG (*n* = 3,935 pairs). All exposures, covariables, outcomes, and additional informative variables were included in the imputation model (see Web Tables 1 and 2, available at https://academic.oup.com/aje, for details on included variables). A separate imputation model including the same variables was fitted for all mother-daughter pairs with data on either daughter’s age at thelarche or daughter’s age at pubarche (our secondary outcomes) and maternal prepregnancy BMI or GWG (*n* = 2,942). We used switching regression in Stata (StataCorp LP, College Station, Texas) as described by Royston (38). We carried out 20 cycles of regression switching and generated 20 imputation data sets. The main analysis results for the 3,935 or 2,942 mother-daughter pairs were obtained by averaging across the results from each of these 20 data sets using Rubin and Schenker’s rules (36).

## RESULTS

Mean age at menarche in the ALSPAC girls was 12.6 (standard deviation (SD), 1.2) years. Early menarche (younger menarcheal age) was less than 11.5 years (1 SD below the mean; 15.6% of the sample). Late menarche (older menarcheal age) was more than 13.8 years (1 SD above the mean; 16.4% of the sample). Mean ages at thelarche and pubarche were 10.15 (SD, 0.03) years and 10.74 (SD, 0.03) years, respectively. The distributions of variables were similar between all singleton female offspring in ALSPAC and the observed and imputed data for eligible participants (see Web Tables 1 and 2).

Table 1 shows the distribution of participant characteristics by daughter’s age at menarche. Maternal prepregnancy BMI, GWG, the probability of smoking during pregnancy, the probability of being nonwhite, and daughter’s prepubertal BMI decreased across categories of daughter’s age at menarche. Maternal age at menarche, maternal age at delivery, daughter’s ages at pubarche and thelarche, and birth weight increased across the age-at-menarche categories. There was no clear pattern of parity or socioeconomic status across categories of daughter’s age at menarche. Similar distributions were observed in complete-case data (Web Table 3).

**Table 1.** Characteristics of Participants According to Daughter’s Age at Menarche in Imputed Data Sets, Avon Longitudinal Study of Parents and Children (*n* = 3,935 Pairs), 1991–2008

Characteristic	Timing of Daughter’s Menarche								
	Early (Age <11.5 Years) (*n* = 615)			Average (Ages 11.5–13.8 Years) (*n* = 2,676)			Late (Age >13.8 Years) (*n* = 644)		
	No. of Pairs	%	Mean (SE)	No. of Pairs	%	Mean (SE)	No. of Pairs	%	Mean (SE)
Prepregnancy BMI^a^			23.8 (0.17)			23.0 (0.08)			22.3 (0.13)
Prepregnancy BMI category									
<18.5 (underweight)	26	4.2		133	5.0		45	7.0	
18.5–24.9 (normal)	407	66.2		1,960	73.2		510	79.1	
25.0–29.9 (overweight)	130	21.2		421	15.7		70	10.9	
≥30.0 (obese)	52	8.4		162	6.0		19	3.0	
Gestational weight gain, kg			12.9 (0.18)			12.5 (0.09)			12.3 (0.18)
IOM gestational weight gain category									
Inadequate	176	28.5		923	34.5		250	38.9	
Adequate	246	40.1		1,010	37.8		253	39.2	
Excessive	193	31.4		743	27.8		141	21.9	
Age at delivery, years			28.3 (0.19)			28.8 (0.09)			29.0 (0.18)
Maternal age at menarche, years			12.3 (0.06)			12.8 (0.03)			13.4 (0.06)
Parity									
0	320	52.1		1,244	46.5		281	43.6	
1	193	31.3		959	35.8		262	40.7	
2	67	10.9		352	13.2		77	11.9	
≥3	35	5.8		121	4.5		24	3.8	
Manual social class^b,c^									
0	505	82.2		2,260	84.5		532	82.6	
1	110	17.8		416	15.5		112	17.4	
Smoking^c^									
Never smoker	444	72.2		2,177	81.3		546	84.8	
Ever smoker	171	27.8		499	18.7		98	15.2	
Daughter’s birth weight, g			3,359.6 (21.19)			3,373.6 (9.61)			3,429.0 (19.55)
Daughter’s prepubertal BMI			17.4 (0.10)			16.4 (0.04)			15.5 (0.07)
Daughter’s ethnicity									
White	569	92.5		2,574	96.2		623	96.8	
Nonwhite	46	7.5		102	3.8		21	3.2	
Daughter’s age at thelarche, years			9.1 (0.05)			10.1 (0.03)			11.3 (0.06)
Daughter’s age at pubarche, years			9.8 (0.06)			10.8 (0.03)			11.7 (0.07)

Abbreviations: BMI, body mass index; IOM, Institute of Medicine; SE, standard error.

^a^ Weight (kg)/height (m)^2^.

^b^ Manual social class was coded as 1 and was defined as including skilled (manual), semiskilled, and unskilled occupations.

^c^ Smoking and socioeconomic status have been dichotomized for presentation.

There was no strong statistical evidence to suggest departure from a linear relationship between maternal prepregnancy BMI or GWG and daughter’s age at menarche, thelarche, or pubarche (all *P*’s > 0.1). There was also no evidence that the association between maternal prepregnancy BMI or GWG and all outcomes differed according to occupational social class (manual/nonmanual) (all *P*’s > 0.5). Prepregnancy BMI and GWG were inversely associated with daughter’s menarcheal age in both the age-adjusted and confounder-adjusted models (Table 2). When birth weight and gestational age were accounted for (model 3), the direct effects remained virtually unchanged in comparison with the estimate in model 2, but there was evidence of a modest positive indirect effect. However, when daughter’s prepubertal BMI was included (model 4), the direct effect was attenuated toward the null value, and there was evidence of negative indirect effects via prepubertal BMI.

**Table 2.** Associations of Maternal Prepregnancy Body Mass Index and Gestational Weight Gain With Daughter’s Ages at Menarche, Thelarche, and Pubarche, Avon Longitudinal Study of Parents and Children, 1991–2008

Outcome Variable and Predictor	No. of Pairs	Model 1^a^		Model 2^b^		Model 3^c^		Model 4^d^	
		β	95% CI	β	95% CI	β	95% CI	β	95% CI
Daughter’s age at menarche									
Prepregnancy BMI^e^	3,935								
Total/direct effect^f^		−0.49	−0.60, −0.37	−0.34	−0.45, −0.22	−0.36	−0.48, −0.24	−0.09	−0.20, 0.03
Indirect effect						0.02	0.01, 0.04	−0.25	−0.30, −0.21
Gestational weight gain, kg	3,935								
Total/direct effect		−0.12	−0.21, −0.02	−0.17	−0.26, −0.07	−0.22	−0.32, −0.12	−0.09	−0.19, 0.004
Indirect effect						0.06	0.03, 0.08	−0.08	−0.10, −0.05
Daughter’s age at thelarche									
Prepregnancy BMI	2,942								
Total/direct effect		−0.92	−1.08, −0.76	−0.77	−0.93, −0.60	−0.77	−0.93, −0.60	−0.37	−0.54, −0.21
Indirect effect						0.002	−0.02, 0.03	−0.39	−0.58, −0.21
Gestational weight gain, kg	2,942								
Total/direct effect		−0.20	−0.34, −0.06	−0.28	−0.42, −0.14	−0.30	−0.45, −0.16	−0.16	−0.30, −0.02
Indirect effect						0.03	−0.01, 0.06	−0.12	−0.19, −0.05
Daughter’s age at pubarche									
Prepregnancy BMI	2,942								
Total/direct effect		−0.38	−0.57, −0.20	−0.25	−0.43, −0.06	−0.25	−0.43, −0.07	−0.09	−0.27, 0.09
Indirect effect						0.004	−0.02, 0.03	−0.15	−0.25, −0.05
Gestational weight gain, kg	2,942								
Total/direct effect		−0.25	−0.40, −0.09	−0.28	−0.43, −0.12	−0.30	−0.47, −0.14	−0.23	−0.39, −0.08
Indirect effect						0.03	−0.01, 0.07	−0.04	−0.08, −0.01

Abbreviations: BMI, body mass index; CI, confidence interval.

^a^ Model 1: Results were adjusted for maternal age and daughter’s ethnicity.

^b^ Model 2: Results were adjusted as in model 1, plus maternal age, parity, maternal smoking during pregnancy, socioeconomic status, and maternal age at menarche. The gestational weight gain model also adjusted for maternal prepregnancy BMI and gestational age.

^c^ Model 3: Results were adjusted as in model 2, plus birth weight and gestational age.

^d^ Model 4: Results were adjusted as in model 2, plus prepubertal BMI.

^e^ Weight (kg)/height (m)^2^.

^f^ The estimates represent total effects in confounder-adjusted models (models 1 and 2) and direct effects in models with mediators included (models 3 and 4).

Prepregnancy BMI and GWG were also inversely associated with ages at thelarche and pubarche (Table 2). For both, the direct effect remained unchanged when birth weight and gestational age were included (model 3). The associations with age at thelarche were substantially attenuated toward the null in model 4 when daughter’s prepubertal BMI was accounted for, with strong evidence of negative indirect effects via prepubertal BMI. A similar pattern was observed for age at pubarche, but the indirect effect accounted for a smaller proportion of the total effect. Sensitivity analysis including only term pregnancies for age at menarche (*n* = 3,606) and age at pubarche or thelarche (*n* = 2,700) yielded results similar to those presented here.

**Figure 2.**
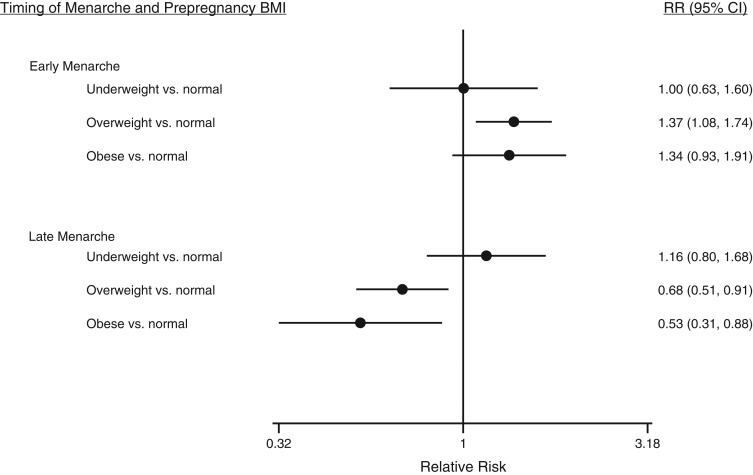
Relative risks (RRs) of early and late menarche (vs. average menarcheal age) according to maternal prepregnancy body mass index (BMI; weight (kg)/height (m)^2^) in the Avon Longitudinal Study of Parents and Children (*n* = 3,935 pairs), 1991–2008. Results were adjusted for maternal age, ethnicity, parity, smoking, socioeconomic status, and maternal age at menarche. Bars, 95% confidence intervals (CIs).

**Figure 3.**
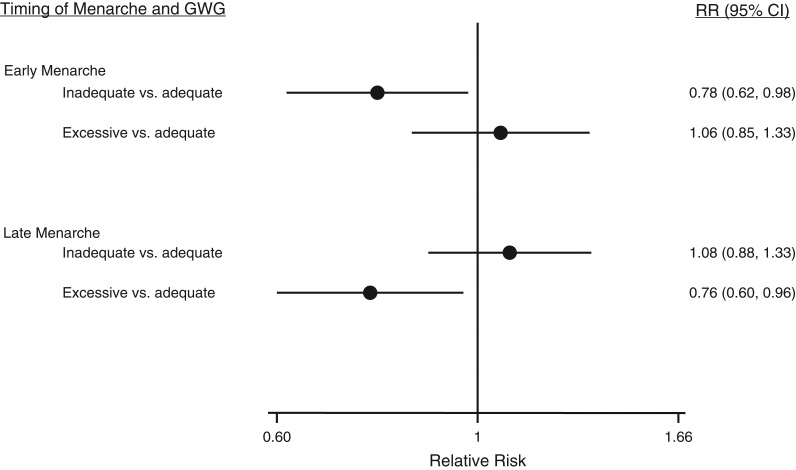
Relative risks (RRs) of early and late menarche (vs. average menarcheal age) according to maternal gestational weight gain (GWG) in the Avon Longitudinal Study of Parents and Children (*n* = 3,935 pairs), 1991–2008. Results were adjusted for maternal age, ethnicity, parity, smoking, socioeconomic status, maternal age at menarche, and gestational age. Bars, 95% confidence intervals (CIs).

Figures 2 and 3 present the confounder-adjusted relative risks of early and late menarche according to categories of maternal prepregnancy BMI and GWG, respectively. Results from all models, including those for continuous BMI and GWG, are provided in Web Table 4. There was no strong evidence of associations of maternal underweight with early or late daughter’s menarcheal age as compared with maternal normal BMI. Maternal overweight was associated with increased risk of early menarche and a lower risk of late menarche. Maternal obesity was associated with a decreased risk of late menarche. Inadequate maternal GWG was associated with a greater risk of early menarche but not late menarche compared with average age at menarche. Maternal excessive GWG was associated with daughter’s late menarche but not early menarche. Greater BMI and, more weakly, GWG were associated with increased risk of early menarche and greater BMI with decreased risk of late menarche in both the age-adjusted and confounder-adjusted models (Web Table 4).

Maternal prepregnancy weight and GWG in gestational weeks 28 and above were inversely associated with daughter’s menarcheal age, while associations between GWG in early pregnancy (≤18 weeks) and midpregnancy (18–28 weeks) and daughter’s age at menarche were consistent with the null (Table 3). However, there was no strong statistical evidence to suggest that associations differed by period of GWG (95% confidence intervals overlapped). Maternal prepregnancy weight and GWG in gestational weeks ≤18 and ≥28 were inversely associated with daughter’s age at thelarche but not GWG between 18 and 28 weeks. There was no strong evidence that GWG in any period of pregnancy was associated with age at pubarche (Table 3).

**Table 3.** Associations Between Maternal Prepregnancy Weight and Gestational Weight Gain in Different Periods of Pregnancy and Daughter’s Ages at Menarche, Thelarche, and Pubarche, Avon Longitudinal Study of Parents and Children, 1991–2008

Outcome Variable and Pregnancy Period^a^	No. of Pairs	Model 1^b^		Model 2^c^	
		β	95% CI	β	95% CI
Daughter’s age at menarche	3,935				
Prepregnancy weight, kg		−0.13	−0.17, −0.09	−0.09	−0.13, −0.06
≤18 WG, kg/week		−1.82	−4.96, 1.32	−1.47	−4.51, 1.58
18–28 WG, kg/week		0.48	−3.45, 4.41	0.39	−3.42, 4.19
≥28 WG, kg/week		−3.81	−6.96, −0.67	−3.95	−7.04, −0.86
Daughter’s age at thelarche	2,942				
Prepregnancy weight, kg		−0.24	−0.30, −0.18	−0.20	−0.26, −0.14
≤18 WG, kg/week		−5.33	−9.79, −0.87	−5.94	−10.36, −1.52
18–28 WG, kg/week		2.80	−2.73, 8.33	3.39	−2.12, 8.89
≥28 WG, kg/week		−6.78	−11.12, −2.44	−6.52	−10.89, −2.15
Daughter’s age at pubarche	2,942				
Prepregnancy weight, kg		−0.09	−0.16, −0.03	−0.06	−0.12, 0.003
≤18 WG, kg/week		−3.87	−8.66, 0.93	−4.24	−8.95, 0.48
18–28 WG, kg/week		−1.81	−7.91, 4.29	−0.87	−6.95, 5.20
≥28 WG, kg/week		−2.89	−7.47, 1.70	−2.89	−7.43, 1.65

Abbreviations: CI, confidence interval; WG, weeks of gestation.

^a^ Defined on the basis of knot points at 18 and 28 weeks (37).

^b^ Model 1: Results were adjusted for maternal age, daughter’s ethnicity, prepregnancy weight, and weight gain during other periods of pregnancy.

^c^ Model 2: Results were adjusted as in model 1, plus parity, maternal smoking during pregnancy, socioeconomic status, and maternal age at menarche.

Results of analyses using the complete-case data (*n* = 2,086) for age at menarche are presented in Web Tables 5–7 and were comparable to the main results obtained using imputed data sets.

## DISCUSSION

In this prospective study of mother-daughter pairs from ALSPAC, we found inverse linear associations between maternal prepregnancy BMI and GWG and daughter’s ages at menarche, thelarche, and pubarche after adjustment for potential confounders, including maternal menarcheal age. This pattern of inverse associations was consistent when BMI, GWG, and menarcheal age were assessed as continuous variables and when they were all assessed as categorical variables. These associations were mediated, though not entirely, by daughter’s own prepubertal BMI.

The inverse linear associations of maternal prepregnancy BMI and GWG (13) with daughter’s age at menarche are consistent with some previous reports (13, 22, 23) but not all. Boynton-Jarrett et al. (11) found evidence of a nonlinear association of GWG with daughter’s age at menarche, such that women whose mothers reported GWG below 10 pounds (4.5 kg) or above 40 pounds (18.2 kg) were 30% more likely to report early menarche. Other investigators found no strong evidence of associations (23–25). It is possible that differences are due to different assessment methods and definitions of GWG and menarcheal age. For example, here we used prospective repeat measures of GWG, while Boynton-Jarrett et al. relied on self-reported data (11). Additionally, various studies have defined late menarche differently: as >13 years (22), 14–16 years (13), or >15 years (11).

**Figure 4.**
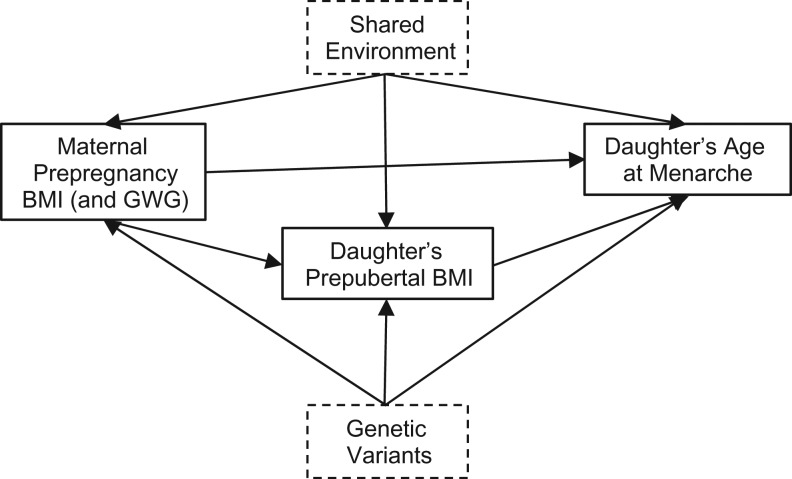
Possible pathways for the observed associations between maternal prepregnancy body mass index (BMI; weight (kg)/height (m)^2^) and gestational weight gain (GWG) and daughter’s age at menarche, Avon Longitudinal Study of Parents and Children (*n* = 3,935 pairs), 1991–2008.

A direct intrauterine effect of maternal prepregnancy BMI and GWG on daughter’s puberty is one possible pathway (Figure 4) by which maternal adiposity may affect a daughter’s pubertal age, perhaps due to exposure to high levels of maternal and/or daughters’ own leptin and other endocrine hormones (7, 24, 39–41). Adiposity and menarcheal age share common genetic determinants (12), and genetic heritability is therefore likely to explain at least part of the observed association between maternal prepregnancy BMI and daughter’s age at menarche, directly and/or via daughter’s prepubertal BMI (25). Although we accounted for maternal age at menarche in analyses, it is unlikely that we captured genetic variation in its entirety. Shared familial environmental factors, such as diet and other health-related behaviors, may also explain the observed association and our finding that daughters’ own prepubertal BMIs mediated the association of interest.

In contrast to our results, other studies of menarcheal age have not found evidence of mediation by daughter’s prepubertal BMI (13, 22). It has been postulated that offspring adiposity in fetal or early life is a more important mediator of the relationship between maternal BMI and daughter’s menarcheal age (42), but this was not the case in our study sample. The largest association appeared to be for GWG in late pregnancy (28 weeks and beyond), though confidence intervals for all periods of pregnancy overlapped. GWG in late pregnancy is mainly driven by fetal growth (30, 43). Mediators of the relationship between maternal adiposity and daughter’s ages at therlarche and pubarche have been less studied; however, prepubertal BMI has been shown to be associated with thelarche onset but not pubarche onset (12). Furthermore, birth weight has not been shown to be associated with entry into Tanner stage 2 (19). Large, well-powered studies could further investigate these relationships.

The present study population comprised a larger sample than those in most previous studies of maternal adiposity and menarcheal age. Thus, these findings contribute to the limited body of literature on the relationship between maternal adiposity and various aspects of pubertal timing. We found similar distributions of characteristics between the mother-offspring pairs included in the study and those lost to follow-up. Furthermore, we used chained equations to impute missing covariable and exposure data and found mostly similar results for the distributions and analysis between complete-case and imputed data. In the present study, age at menarche and Tanner scores were reported on annual questionnaires administered throughout childhood and adolescence, so the lag time between the event and reporting was shorter than that in most studies (11, 22, 23, 25). Differing recall periods can affect accuracy and hence may introduce bias, especially because age at menarche has relatively small variation (44).

Mediation analyses assume no correlated measurement error between exposure and mediator; no unmeasured confounding of the exposure-outcome, mediator-outcome, and exposure-mediator relationships; and no effect of the exposure that confounds the mediator-outcome relationship (45, 46). We used heights and weights measured in duplicate at research clinics using standard procedures to minimize measurement error for daughter’s own prepubertal BMI, and the birth weight measures used were obtained from multiple sources, including obstetrical data. We therefore cannot think of any reason why measurement error in the exposures (maternal prepregnancy BMI and GWG) and these mediators would be correlated. While we adjusted for socioeconomic status, which is likely to account for some shared familial environmental factors, we cannot rule out potential collider bias arising from unmeasured confounding between daughter’s birth weight or prepubertal BMI and daughter’s menarcheal age.

To conclude, we found that greater maternal prepregnancy BMI and GWG are associated with earlier daughters’ ages at menarche, pubarche, and thelarche, even when accounting for mothers’ own menarcheal age and other potential confounders. Some of these associations appear to be mediated by prepubertal BMI. Understanding of pathways and mechanisms affecting puberty is important because of associations of early menarche with important health outcomes such as premature death, cardiovascular disease, and ovarian cancer (7).

## Supplementary Material

Web Material

## Abbreviations

ALSPAC: Avon Longitudinal Study of Parents and Children
BMI: body mass index
GWG: gestational weight gain
SD: standard deviation
