# Supplementary material for: Associations Between Maternal Prepregnancy Body Mass Index and Gestational Weight Gain and Daughter’s Age at Menarche: The Avon Longitudinal Study of Parents and Children
Source: Am J Epidemiol. 2017 Sep 11;187(4):677–86. doi: 10.1093/aje/kwx308 (PMC5888997; doi:10.1093/aje/kwx308)
Supplement: Web Material [file kwx308lawnwebmaterialfinal.pdf]

Web Table 1. Comparison of distributions between imputed and complete-case data sets.

		Entire ALSPAC Cohort of Singleton, Female Offspring (*N* = 6592)		Eligible Sample			Sample Included in the Analysis of the Primary Outcome, Age at Menarche (*N* = 3935)
		N	**% or Mean (Standard Error)**	**No. with Non-Missing Data on Each Variable**	**Observed Data for Eligible Participants (% or Mean (Standard Error))**	**% Imputed**	**Imputed Data, % or Mean (Standard Error)**
Maternal prepregnancy BMI, kg/m^2		5489	22.90 (0.05)	3593	22.90 (0.06)	8.69	22.98 (0.06)
Maternal prepregnancy BMI	underweight	5489	5.37	3593	4.84	8.69	5.21
	normal		74.02		74.45		73.06
	overweight		15.12		15.20		15.83
	obese		5.48		5.51		5.89
Gestational weight gain, kg		5832	12.37 (0.06)	3616	12.54 (0.08)	8.11	12.53 (0.07)
IOM categories of gestational weight gain	inadequate	4935	34.55	3274	34.09	16.8	34.28
	adequate		37.69		37.94		38.35
	excessive		27.76		27.98		27.37
Daughters' age at menarche, months		3991	151.55 (0.22)	3935	151.59 (0.22)	0	
Maternal age at delivery, years		6592	27.85 (0.06)	3935	28.74 (0.07)	0	
Maternal age at menarche, years		5280	12.83 (0.02)	3419	12.82 (0.02)	13.11	12.82 (0.03)

Birth weight, g		6511	3357.64 (6.35)	3883	3381.73 (8.00)	1.32	3380.46 (8.01)
Prepubertal BMI, kg/m^2		3708	16.37 (0.04)	3102	16.35 (0.04)	21.17	16.39 (0.04)
	early	3991	15.71		15.63		
Daughters' age at menarche	average		67.95	3935	68.01	0	
	late		16.34		16.37		
	0	6111	45.46		46.94		46.89
Parity	1		34.77	3779	35.91	3.96	35.92
	2		13.99		12.57		12.60
	3+		5.78		4.58		4.59
Socioeconomic status	non-manual	5464	80.64	3599	84.72	8.54	83.81
	manual		19.36		15.28		16.19
Smoking	never	6199	76.48	3831	80.61	2.64	80.48
	ever		24.52		19.39		19.52
Ethnicity	white	5717	95.00	3726	95.92	5.31	95.70
	non-white		5.00		4.08		4.30
Daughters' age at thelarche		2702	121.76 (0.32)	2450			121.83 (0.29)
Daughters' age at pubarche		2488	129.21 (0.34)	2311			129.09 (0.35)

Note: Smoking and socioeconomic status have been dichotomized for presentation.

In additional to the variables included in Web Table 1, we included the following variables in the imputation model: maternal ethnicity, maternal education, breast feeding status, age at thelarche, age at pubarche, and clinic and/or questionnaire data for daughters' BMI at 6.5, 8, and 9 years.

Web Table 2. Comparison of distributions between age at thelarche and pubarche imputed and complete-case data sets.

		Number of Eligible Participants With Non-Missing Data	% Imputed	Observed Data (% or Mean (Standard Error))	Imputed Data [*N* = 2942] (% or Mean (Standard Error))
Maternal prepregnancy BMI, kg/m^2		2739	6.90	22.80 (0.07)	22.87 (0.07)
Gestational weight gain, kg		2687	8.67	12.60 (0.09)	12.59 (0.08)
Maternal age at delivery, years		2942	0	28.96 (0.08)	
Maternal age at menarche, years		2610	11.28	12.84 (0.03)	12.85 (0.03)
Birth weight, g		2904	1.29	3386.37 (9.07)	3385.52 (9.07)
Prepubertal BMI, kg/m^2		2449	16.76	16.35 (0.04)	16.41 (0.05)
Parity	0	2860	2.79	48.60	48.60
	1			35.14	35.17
	2			12.10	12.08
	3+			4.16	4.16
Socioeconomic status	non-manual	2762	8.54	85.92	85.36
	manual			14.08	14.64
Smoking	never	2891	6.12	82.84	82.74
	ever			17.16	17.26
Ethnicity	white	2834	3.67	96.22	96.08
	non-white			3.78	3.92
Daughters' age at thelarche		2675	9.08	121.79 (0.32)	121.85 (0.32)
Daughters' age at pubarche		2467	16.15	129.20 (0.34)	128.90 (0.32)

Note: Smoking and socioeconomic status have been dichotomized for presentation.

In additional to the variables included in Web table 2, we included the following variables in the imputation model: maternal ethnicity, maternal education, breast feeding status, age at menarche, and clinic and/or questionnaire data for daughters' BMI at 6.5, 8, and 9 years.

Web Table 3. Participant characteristics by daughters' age at menarche in the complete-case data set (*N* = 2086).

		Early Menarche (<11.5 years) (*n* = 323)	Average Menarche (11.5–13.8 years) (*n* = 1424)	Late Menarche (>13.8 years) (*n* = 339)
		Mean (Standard Error) or No. (%)		
Prepregnancy BMI, kg/m^2		23.49 (0.20)	22.83 (0.10)	22.26 (0.18)
Prepregnancy BMI	underweight	13 (4.02)	64 (4.49)	20 (5.90)
	normal	220 (68.11)	1073 (75.35)	273 (80.53)
	overweight	71 (21.98)	220 (15.45)	33 (9.73)
	obese	19 (5.88)	67 (4.71)	13 (3.83)
Gestational weight gain, kg		13.03 (0.23)	12.74 (0.12)	12.21 (0.23)
IOM categories of gestational weight gain	inadequate	80 (24.77)	469 (32.94)	137 (40.41)
	adequate	142 (43.96)	554 (38.90)	130 (38.35)
	excessive	101 (31.27)	401 (28.16)	72 (21.24)
Age at delivery, years		29.10 (0.25)	29.17 (0.11)	29.48 (0.24)
Maternal age at menarche, years		12.27 (0.08)	12.81 (0.04)	13.31 (0.08)
Parity	0	175 (54.18)	692 (48.60)	154 (45.43)
	1	101 (31.27)	511 (35.88)	136 (40.12)
	2	30 (9.29)	165 (11.59)	40 (11.80)
	3+	17 (5.26)	56 (3.93)	9 (2.65)
Manual social class	0	289 (89.47)	1247 (87.57)	290 (85.55)
	1	34 (10.53)	177 (12.43)	49 (14.45)
Smoking	never	253 (78.33)	1197 (84.06)	286 (87.02)
	ever	70 (21.67)	227 (15.94)	43 (12.98)
Daughters' birth weight, g		3425.77 (26.85)	3426.21 (11.34)	3487.84 (24.00)
Daughters' prepubertal BMI, kg/m^2		17.46 (0.13)	16.31 (0.05)	15.54 (0.09)
Ethnicity	white	298 (92.26)	1376 (96.63)	319 (97.05)
	non-white	25 (7.74)	48 (3.37)	10 (2.95)

Note: Smoking and socioeconomic status have been dichotomized for presentation.

Web Table 4. Early and late menarche relative risk ratios (vs. average menarcheal age) for maternal underweight, overweight and obese prepregnancy BMI (vs. normal weight) and early and late menarche odds ratios (vs. average menarcheal age) for maternal inadequate and excessive gestational weight gain (vs. adequate) (*N* = 3935).

	Model 1	Model 2	Model 3	Model 4
	RR (95% CI)			
**Prepregnancy BMI, kg/m^2**				
**Early menarche:**				
Underweight vs. normal	0.90 (0.57, 1.42)	1.00 (0.63, 1.60)	1.00 (0.63, 1.59)	1.12 (0.70, 1.79)
Overweight vs. normal	1.49 (1.18, 1.88)	1.37 (1.08, 1.74)	1.38 (1.09, 1.75)	1.18 (0.92, 1.51)
Obese vs. normal	1.54 (1.10, 2.16)	1.34 (0.93, 1.91)	1.35 (0.94, 1.94)	0.97 (0.66, 1.42)
Per 1 BMI unit	1.05 (1.03, 1.07)	1.04 (1.02, 1.06)	1.04 (1.02, 1.06)	1.01 (0.99, 1.04)
**Late menarche:**				
Underweight vs. normal	1.31 (0.91, 1.89)	1.16 (0.80, 1.68)	1.22 (0.84, 1.77)	0.96 (0.66, 1.41)
Overweight vs. normal	0.64 (0.48, 0.85)	0.68 (0.51, 0.91)	0.66 (0.50, 0.89)	0.80 (0.60, 1.07)
Obese vs. normal	0.46 (0.28, 0.76)	0.53 (0.31, 0.88)	0.51 (0.30, 0.85)	0.71 (0.42, 1.20)
Per 1 BMI unit	0.94 (0.92, 0.97)	0.96 (0.93, 0.98)	0.95 (0.93, 0.98)	0.99 (0.96, 1.02)
**Gestational weight gain, kg**				
**Early menarche:**				
Inadequate vs. adequate	0.78 (0.62, 0.98)	0.78 (0.62, 0.99)	0.77 (0.61, 0.98)	0.83 (0.65, 1.05)
Excessive vs. adequate	1.06 (0.85, 1.33)	1.01 (0.81, 1.27)	1.03 (0.82, 1.30)	0.91 (0.72, 1.16)
Per 1 kg	1.02 (1.00, 1.04)	1.02 (1.00, 1.04)	1.03 (1.01, 1.05)	1.01 (0.99, 1.04)
**Late menarche:**				
Inadequate vs. adequate	1.08 (0.88, 1.33)	1.08 (0.88, 1.34)	1.14 (0.92, 1.41)	1.02 (0.83, 1.26)
Excessive vs. adequate	0.76 (0.60, 0.96)	0.79 (0.62, 1.00)	0.75 (0.59, 0.96)	0.87 (0.68, 1.10)
Per 1 kg	0.99 (0.97, 1.01)	0.98 (0.96, 1.00)	0.97 (0.95, 0.99)	0.99 (0.97, 1.01)

Model 1 - adjusted for maternal age and ethnicity.

Model 2 - as in model 1 plus, parity, maternal smoking during pregnancy, socioeconomic status and maternal age at menarche.

The GWG model also adjusted gestational age. The GWG per 1 kg also adjusted for maternal prepregnancy BMI.

Model 3 – as in model 2 plus birth weight and gestational age.

Model 4 – as in model 2 plus prepubertal BMI.

Web Table 5. Associations between maternal prepregnancy BMI, gestational weight gain and daughters' age at menarche using complete-case data (*N* = 2086).

		Model 1	Model 2	Model 3	Model 4
		Mean Difference (95% CI)			
**Prepregnancy BMI, kg/m^2**	Total/direct effect*	-0.40 (-0.57, -0.24)	-0.26 (-0.42, -0.10)	-0.29 (-0.45, -0.13)	0.04 (-0.13, 0.20)
	Indirect effect			0.03 (0.003, 0.06)	-0.30 (-0.37, -0.22)
**Gestational weight gain, kg**	Total/direct effect*	-0.19 (-0.33, -0.05)	-0.21 (-0.34, -0.07)	-0.27 (-0.41, -0.13)	-0.13 (-0.27, -0.003)
	Indirect effect			0.07 (0.03, 0.11)	-0.07 (-0.11, -0.04)

Model 1- adjusted for maternal age and ethnicity.

Model 2 - as in model 1 plus maternal age, parity, maternal smoking during pregnancy, socioeconomic status and maternal age at menarche. The GWG model also adjusted for maternal prepregnancy BMI and gestational age.

Model 3 – as in model 2 plus birth weight and gestational age.

Model 4 – as in model 2 plus prepubertal BMI.

* The estimates represent total effects in confounder adjusted models (models 1 and 2) and direct effects in models with mediators included (models 3 and 4).

Web Table 6. Associations between maternal GWG in different periods of pregnancy and daughters' age at menarche in complete-case data (*N* = 2086)

**Gestational Weight Gain**	**Model 1**	**Model 2**
0 weeks, kg	-0.09 (-0.15, -0.03)	-0.05 (-0.11, 0.003)
0-18 weeks, kg/week	0.17 (-4.05, 4.38)	0.95 (-3.12, 5.03)
18-28 weeks, kg/week	-3.23 (-8.63, 2.16)	-3.43 (-8.66, 1.79)
28+ weeks, kg/week	-3.09 (-7.26, 1.09)	-2.92 (-6.98, 1.15)

Model 1 - adjusted for maternal age, ethnicity, weight at week 0 and weight gain in other periods of pregnancy.

Model 2 - as in model 1 plus parity, maternal smoking during pregnancy, socioeconomic status, and maternal age at menarche.

Model 3 – as in model 2 plus birth weight.

Model 4 – as in model 2 plus prepubertal BMI.

Web Table 7. Early and late menarche relative risk ratios (vs. average menarcheal age) for maternal underweight, overweight, and obese prepregnancy BMI (vs. normal weight) and early and late menarche odds ratios (vs. average menarcheal age) for maternal inadequate and excessive gestational weight gain (vs. adequate) in complete-case data (*N* = 2086).

	**Model 1**	**Model 2**	**Model 3**	**Model 4**
		**RR (95% CI)**		
**Prepregnancy BMI, kg/m^2**				
**Early menarche:**				
Underweight vs. normal	1.02 (0.55, 1.89)	1.22 (0.65, 2.28)	1.23 (0.66, 2.30)	1.38 (0.73, 2.62)
Overweight vs. normal	1.58 (1.16, 2.14)	1.47 (1.07, 2.01)	1.44 (1.05, 1.99)	1.21 (0.87, 1.68)
Obese vs. normal	1.44 (0.85, 2.45)	1.30 (0.75, 2.24)	1.27 (0.73, 2.21)	0.83 (0.47, 1.48)
Per 1 BMI unit	1.05 (1.02, 1.08)	1.04 (1.00, 1.07)	1.04 (1.00, 1.07)	1.00 (0.97, 1.04)
**Late menarche:**				
Underweight vs. normal	1.24 (0.74, 2.08)	1.11 (0.65, 1.88)	1.18 (0.70, 2.02)	0.93 (0.55, 1.60)
Overweight vs. normal	0.59 (0.40, 0.88)	0.63 (0.43, 0.94)	0.61 (0.41, 0.90)	0.73 (0.49, 1.08)
Obese vs. normal	0.76 (0.42, 1.41)	0.88 (0.47, 1.65)	0.85 (0.46, 1.59)	1.27 (0.67, 2.42)
Per 1 BMI unit	0.95 (0.92, 0.99)	0.96 (0.93, 1.00)	0.96 (0.92, 0.99)	1.00 (0.96, 1.03)
**Gestational weight gain, kg**				
**Early menarche:**				
Inadequate vs. adequate	0.66 (0.49, 0.89)	0.68 (0.50, 0.93)	0.69 (0.51, 0.95)	0.70 (0.52, 0.96)
Excessive vs. adequate	0.99 (0.74, 1.32)	0.95 (0.71, 1.27)	0.94 (0.70, 1.26)	0.83 (0.61, 1.12)
Per 1 kg	1.02 (0.99, 1.05)	1.02 (0.99, 1.05)	1.02 (0.99, 1.05)	1.01 (0.98, 1.04)
**Late menarche:**				
Inadequate vs. adequate	1.24 (0.95, 1.62)	1.24 (0.94, 1.63)	1.34 (1.01, 1.77)	1.18 (0.89, 1.56)
Excessive vs. adequate	0.77 (0.56, 1.06)	0.80 (0.58, 1.11)	0.77 (0.55, 1.06)	0.88 (0.64, 1.22)
Per 1 kg	0.97 (0.95, 1.00)	0.97 (0.94, 1.00)	0.95 (0.92, 0.98)	0.97 (0.95, 1.00)

Model 1- adjusted for maternal age and ethnicity.
Model 2 - as in model 1 plus, parity, maternal smoking during pregnancy, socioeconomic status, and maternal age at menarche. The GWG model also adjusted for gestational age.
Model 3 – as in model 2 plus birth weight and gestational age.
Model 4 – as in model 2 plus prepubertal BMI.
